# Comparing needle types and aspiration techniques in EUS-TA to optimize diagnostic efficacy and specimen quality in patients with pancreatic lesions

**DOI:** 10.3389/fmed.2024.1422600

**Published:** 2024-12-06

**Authors:** Rumin Shang, Xiangming Han, Fangwei He, Lihua Huang, Cui Zeng, Kun Chen, Fei Lv, Xiangwu Ding

**Affiliations:** ^1^Department of Gastroenterology, Wuhan Pu’ai Hospital, Tongji Medical College, Huazhong University of Science and Technology, Wuhan, China; ^2^Department of Oncology, Nanjing Drum Tower Hospital, Nanjing, China; ^3^Department of Gastroenterology, Wuhan Fourth Hospital, Wuhan, China; ^4^Department of Pathology, Wuhan Fourth Hospital, Wuhan, China

**Keywords:** endoscopic ultrasound-guided tissue acquisition, biopsy needle, suction technique, heparin, diagnosis, histological integrity, blood cell contamination

## Abstract

**Purpose:**

In solid pancreatic lesions (SPLs), we compared the diagnostic efficacy of a 19G fine-needle aspiration (FNA) needle and a 22G ProCore fine-needle biopsy (FNB) needle, We also compared the specimen quality between the standard suction (SS) technique and heparinized wet-suction (HWS) technique.

**Methods:**

All cases of endoscopic ultrasound-guided tissue acquisition (EUS-TA) by 19G FNA or 22G FNB for SPLs in a single-centre hospital were retrospectively reviewed. The diagnostic yield was compared between the 19G and 22G groups. Univariate and multivariate logistic regression analyses were used to identify optimal factors for a correct histological diagnosis. We also examined tissue integrity, the length of the tissue cores, and the rate of blood cell contamination between the SS and HWS groups.

**Results:**

One hundred seventy-one and sixty-three patients were included in the comparisons of needle types and suction techniques, respectively. The 19G group had higher histological diagnosis rates compared to the 22G group for the first pass (87.8% vs. 70.4%, *p* = 0.005), the second pass (82.2% vs. 65.4%, *p* = 0.012), the first two passes (90.0% vs. 72.8%, *p* = 0.004), and the final diagnosis (91.1% vs. 79%, *p* = 0.025). Through macroscopic on-site evaluation, a significantly higher proportion of patients in the 22G group required a third needle pass compared to the 19G group (88.9% vs. 67.8%, *p* = 0.002). The total procedure time was shorter in the 19G group than in the 22G group (*p* < 0.001). The HWS group showed superiority over the SS group in terms of the total length of tissue cores (*p* < 0.001) and the total length of white tissue cores (*p* = 0.005). The HWS group, compared to the SS group, can enhance the tissue integrity (*p* = 0.024) and reduce blood cell contamination (*p* = 0.040) during the first needle pass. There was no significant difference in complication rates between the needle puncture groups (*p* = 0.770) or the aspiration technique groups (*p* = 0.654).

**Conclusion:**

Compared to the 22G FNB needle, endoscopists should consider using the 19G FNA needle when appropriate. Furthermore, the use of the HWS technique for the first pass is recommended to improve specimen quality.

## 1 Introduction

Endoscopic ultrasound-guided tissue acquisition (EUS-TA) has become the preferred method for the pathological evaluation of solid pancreatic lesions (SPLs) (1, 2). However, its diagnostic efficacy depends on factors such as the size and type of the biopsy needle (3), the suction technique used (4), macroscopic on-site evaluation (MOSE) (5), the number of needle passes (6), and the experience of the endoscopist (7). Although cytological examination under EUS-TA achieves excellent diagnostic accuracy in most cases, diseases such as autoimmune pancreatitis, solid pseudopapillary neoplasm, and neuroendocrine tumors may be difficult to diagnose without ample tissue for immunohistochemical (IHC) analysis (8).

The standard 19G fine-needle aspiration (FNA) needle, with its large caliber, has been proven to obtain histological samples of lesions, achieving a diagnostic accuracy of 94.4% (9). To improve tissue acquisition, fine-needle biopsy (FNB) needles have entered into clinical practice (10). The special tip design of FNB needles are those with either a specially shaped end cutting tip [SharkCore (11, 12) or Franseen (13, 14)], or a side slot type at their distal portion [ProCore (15, 16)]. The European Society of Gastrointestinal Endoscopy (ESGE) recommends the use of 19G FNA needles, 19G FNB needles, or 22G FNB needles for tissue specimen acquisition (17). The 19G FNB needle, due to its large caliber and the special types of cutting tip, tends to produce bloodier samples and causes significant tissue damage, leading to a higher incidence of adverse events such as bleeding and pancreatic fistula (10, 18, 19). Currently, a direct comparison between 19G FNA and 22G FNB in the pancreas is still lacking.

Moreover, the choice of suction technique is related to the quality of the pathological specimen, thereby affecting diagnostic accuracy (10). Common suction techniques in clinical practice include standard suction (SS) (typically using 10 mL negative pressure), slow pull (SP), and wet-suction (WS) techniques. Recent studies indicate that, in liver lesions, the heparinized wet-suction (HWS) technique has shown better tissue acquisition compared to SS technique (20). However, research on this technique in pancreatic lesions is still insufficient. Therefore, further exploration is needed in the choice of needle types and suction techniques. This study aims to compare in SPLs: (1) the diagnostic efficacy of 19G FNA needles versus 22G FNB needles; and (2) the differences in histological integrity, sample volume, and blood contamination between HWS and SS techniques.

## 2 Participants and methods

### 2.1 Participants

We conducted a retrospective cohort single-center study, including patients with SPLs who underwent EUS-TA at Wuhan Fourth Hospital from January 2012 to January 2024 as the research subjects. The reporting of this study followed the Strengthening the Reporting of Observational Studies in Epidemiology (STROBE) statement (Supplementary Table S1). All patients were hospitalized. Clinical data were collected, including patient age, gender, lesion location, and suction technique. The study complied with the Declaration of Helsinki (revised in 2013). It was approved by the Ethics Committees of Wuhan Fourth Hospital (ID: KY 2024-103-01). Inclusion criteria: all patients that underwent an EUS-TA for the characterization of a SPL. Exclusion criteria: (1) pancreatic cystic lesions; (2) coagulation dysfunction: international normalized ratio (INR) >1.5 or platelet count <80,000/mm^3^; (3) patients who used other puncture needles or aspiration techniques; (4) cases with missing basic patient information, puncture operation records, or follow-up data.

### 2.2 EUS-TA procedure

All endoscopic ultrasound procedures for the enrolled patients were performed by a highly experienced endoscopist who performs more than 50 endoscopic ultrasound examinations annually. Linear echoendoscope were used, including the GF-UCT 260 (Olympus^®^, Tokyo, Japan) and the EG 3830UTK (Pentax^®^, Tokyo, Japan). Our center initially used the 19G FNA (Medi-Globe GmbH^®^, Rosenheim, Germany; or COOK Medical^®^, United States) needle but switched to the 22G FNB (22G EchoTip, COOK Medical^®^, United States) needle with the introduction of the EchoTip ProCore in 2017. Clinical experience, however, showed that the 22G FNB provided lower sample integrity and diagnostic accuracy than the 19G FNA. Thus, since 2022, we have primarily returned to using the 19G FNA for pancreatic punctures. Before the procedure, the patient was positioned in the left lateral decubitus position and administered intravenous propofol (initial dose of 2.0–2.5 mg/kg, maintained at 8–10 mg/kg/h). For patients unable to receive intravenous anesthesia due to cardiopulmonary disease or other reasons, intravenous sedation with 5 mg diazepam and analgesia with 50 mg meperidine were provided. Vital signs were continuously monitored throughout the procedure. During the procedure, the stylet was removed prior to puncturing. SS technique and HWS technique were used. After the lesion was identified by EUS (when puncturing the head of the pancreas, the EUS probe is positioned in the duodenum. For punctures of the neck, body, and tail of the pancreas, the probe is positioned in the stomach body), the needle was advanced into the lesion. To optimize each needle pass, a fan-shaped aspiration method was used, and each needle pass involved 20 to 30 actuation (1 actuation means 1 to-and-fro needle movement, quickly advanced and then slowly pulled back) in the lesion. The rapid onsite cytopathology evaluation (ROSE) is unavailable. The number of needle pass was determined by the endoscopist based on the MOSE and experience.

### 2.3 Sample collection and processing

**Figure 1:**
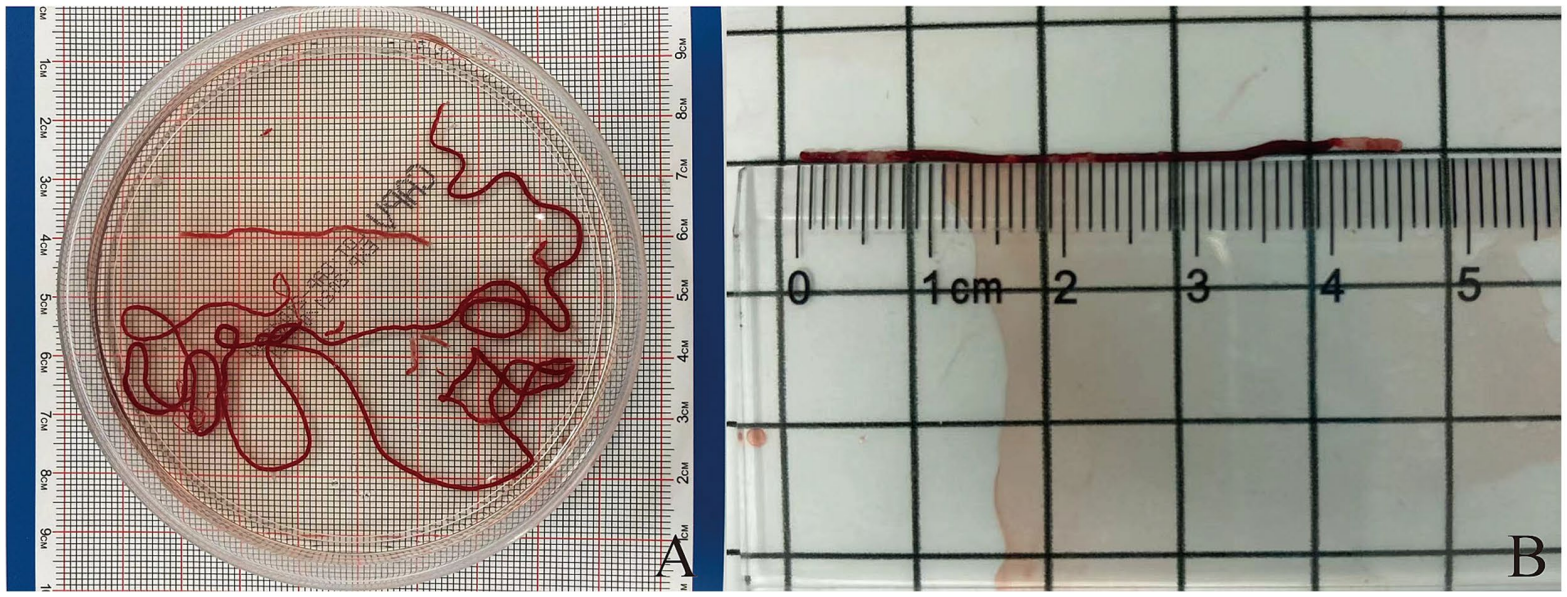
**(A)** The strip of tissue sample is placed into a transparent culture dish containing a 10% neutral buffered formalin solution. **(B)** Measurement of the length of puncture tissue (both red and white parts).

After each pass, the puncture needle is removed from the endoscope, and a stylet is inserted into the needle to push the strip-like tissue sample into a clear petri dish containing 10% neutral buffered formalin solution (Figure 1). Typically, the strip-like tissue samples consist of red parts (mostly blood clots or mixed tissue) and white parts (usually the target tissue) (21) (Figure 1). The endoscopist performs MOSE to determine the presence of significant volumes of white tissue blocks (at least 2 mm of white or pale-yellow tissue was observed) (22). (1) The petri dish is gently and intermittently shaken to prevent blood coagulation. Solid tissue components are embedded in paraffin, sectioned, and stained with hematoxylin-eosin (HE) and IHC staining. (2) Air is drawn into a standard syringe (10 mL), and the residual blood and tissue inside the needle tip are pushed onto a glass slide to prepare a cytological smear, which is then stained with Papanicolaou stain. (3) The saline washout from the syringe and needle containing blood and tissue is collected for liquid-based cytology testing (membrane-based and sedimentation). Samples obtained from different numbers of punctures are stored separately, and each specimen container is labeled in sequence. Histological and cytological samples are independently reviewed and diagnosed by two pathologists. If there is a discrepancy in the diagnosis, a final determination is made by a pathology quality control expert. A ruler (with measurements accurate to 1 millimeter) is used to measure the total length of biopsy tissue strips and the length of white core tissue strips from the first, second, and third punctures.

### 2.4 Result interpretation

#### 2.4.1 Cytological and histological evaluation criteria

The reports will be stratified into five diagnostic categories for histological and cytological evaluation: “insufficient,” “positive for malignancy,” “suspicious for malignancy,” “atypia,” and “negative for malignancy.” A diagnosis of “positive for malignancy” will be made if the reports contain terms such as “diagnostic for malignancy,” “compatible with carcinoma,” “consistent with adenocarcinoma,” “positive for malignant cells,” “malignant cells present,” or when specified by the exact tumor type. The category “positive for malignancy” will not be considered if the reports contain terms such as “suspicious for malignancy,” “atypia,” or “negative for malignancy.”

#### 2.4.2 Histological integrity assessment

**Figure 2:**
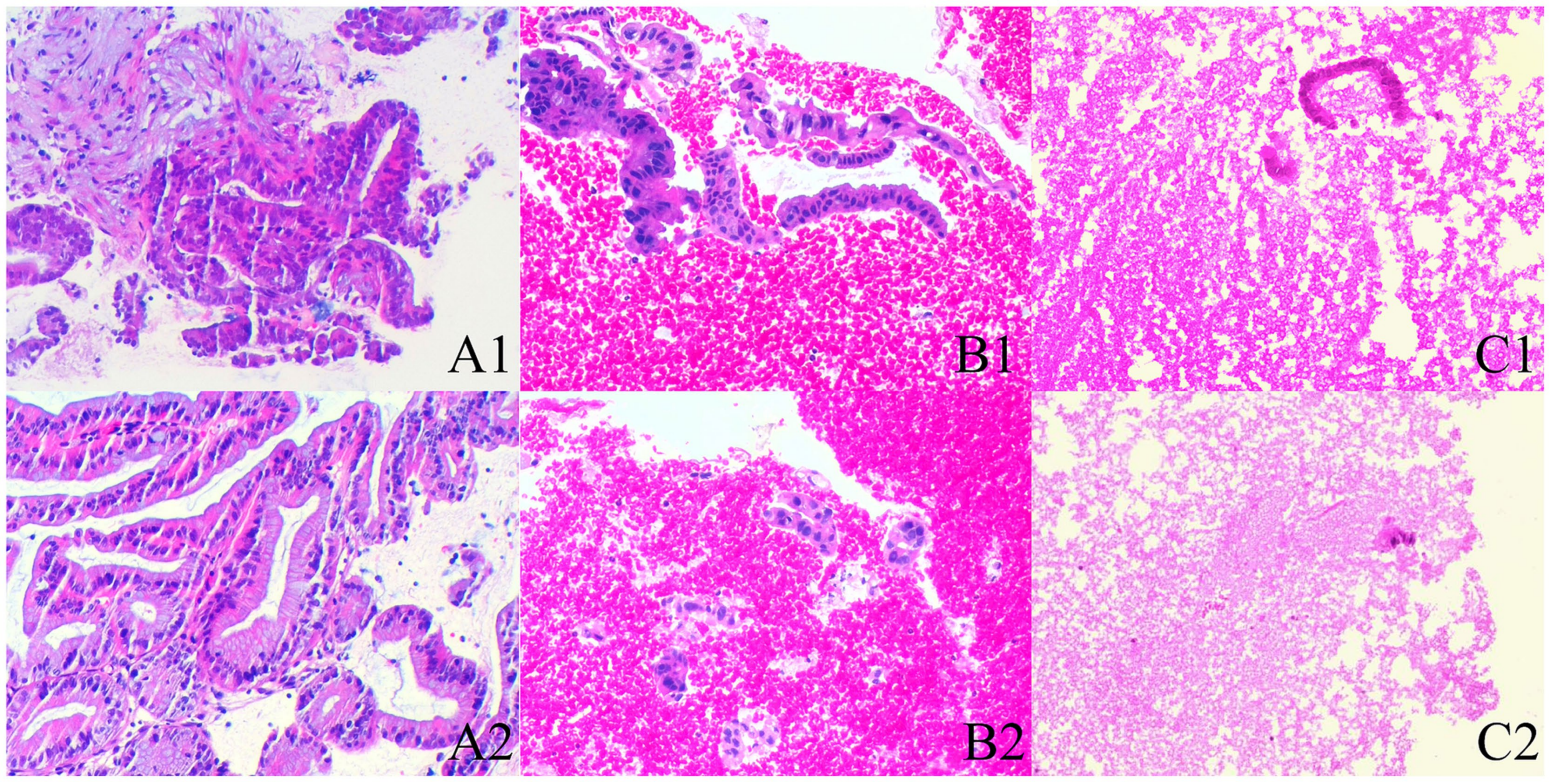
The histological integrity of the samples (HE stain; magnification, ×100). **(A1,A2)** High quality. Histological cores are present, with tumor cells arranged in a glandular pattern, showing partial gland fusion and prominent malignant accompanied by significant stromal reaction, diagnosed as pancreatic carcinoma (**A1,A2** are derived from the same patient). **(B1,B2)** Low quality. Atypical glands are distributed sporadically with rupture, and no fibrous stroma is observed; however, the presence of malignant cells allows for a diagnosis of pancreatic carcinoma based on cellular morphology (**B1,B2** are derived from the same patient). **(C1,C2)** Insufficient material. The specimen predominantly shows hemorrhage, with only a few normal glandular epithelial cells present, preventing a definitive diagnosis (**C1,C2** are derived from the same patient).

Histological integrity is classified into three levels (23) (Figure 2): high quality: presence of core tissue (defined as structurally intact tissue blocks with a longitudinal axis of at least 550 μm under high magnification), with clear lesion characteristics sufficient for diagnosis. Low quality: presence of core fragments, although not meeting the criteria for structural integrity, diagnosis can still be made based on cellular morphology. Insufficient: failure to obtain lesion tissue or inability to make a diagnosis based on the obtained sample, in conjunction with cytopathological classification.

#### 2.4.3 Blood cell contamination grading

**Figure 3:**
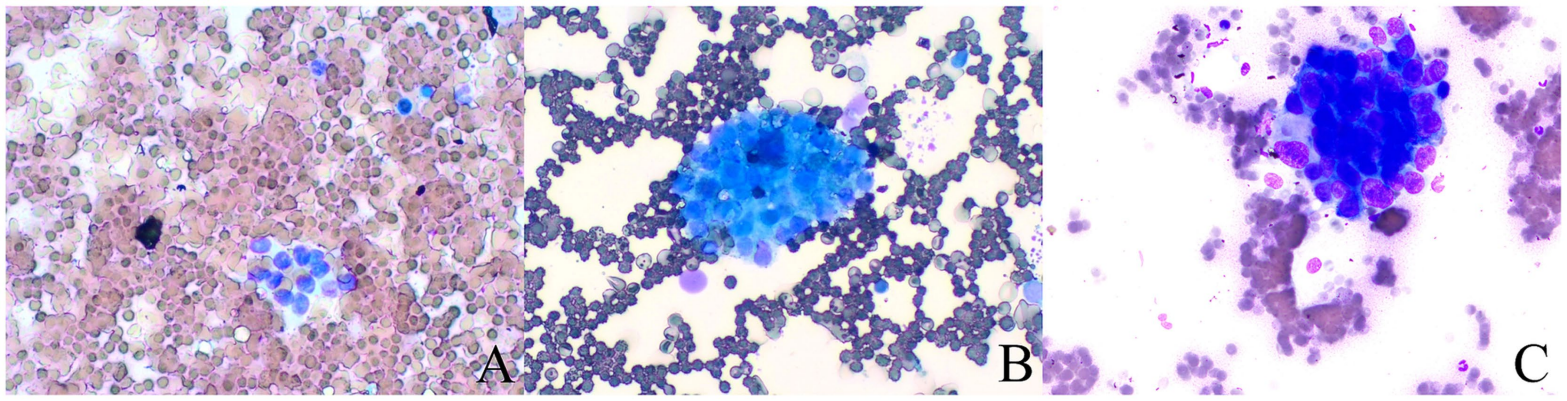
The grading of blood cell contamination (Papanicolaou stain; magnification, ×100). **(A)** Microscopic examination reveals some normal pancreatic epithelial cells, significant blood contamination, and cellular aggregation or layering. **(B)** Microscopic examination shows some normal pancreatic epithelial cells along with a small number of lymphocytes and moderate blood contamination. **(C)** Cancer cells are round to oval, with relatively large nuclei and prominent nucleoli, displaying strong adhesion and forming clusters. There is mild blood contamination with sparse red blood cells.

The grading of blood cell contamination in direct smears is divided into three levels (24) (Figure 3): minimal: contamination area is less than 25% of the smear surface area. Moderate: contamination area is 25–50% of the smear surface area. Significant: contamination area is more than 50% of the smear surface area.

### 2.5 Follow-up

All patients were hospitalized. Within 48 h after the puncture, routine blood tests, serum amylase levels, clinical symptoms, and signs are monitored. Complications, including but not limited to pancreatitis, abdominal pain, fever, gastrointestinal bleeding, and pancreatic leakage, are identified. A phone follow-up is conducted after 1 week. We also conducted clinical follow-ups at 6 months post-discharge. The final diagnostic criteria are as follows: for patients who underwent surgery, follow-up was completed once a definitive histopathological diagnosis was obtained from the surgical specimen. In cases where no surgical pathology was available, lesions were considered benign if they resolved spontaneously or if imaging follow-up revealed no signs of progression. However, if clinical or imaging assessments demonstrated lesion progression or metastasis, accompanied by malignant symptoms such as weight loss, anemia, or death, the lesion was classified as malignant.

### 2.6 Statistical analysis

Continuous variables are first tested for normality. For variables that follow a normal distribution, the mean ± standard deviation (mean, SD) is used for presentation, while for non-normally distributed variables, the median (P25, P75) is employed. The *t*-test or Wilcoxon rank-sum test is used for analysis. Categorical variables are expressed as counts and percentages and analyzed using the chi-square test. A *p*-value of <0.05 is considered statistically significant. All statistical analyses are performed using IBM SPSS V26.0 software.

## 3 Results

### 3.1 Patient characteristics and final diagnosis

In this study, 237 patients with pancreatic lesions who underwent puncture were initially included. Of these, 36 were excluded due to pancreatic cystic lesions and 30 were excluded due to incomplete puncture data, leaving 171 patients for the analysis of the type of puncture needle. Among these, 38 had fewer than three needle aspirations, 23 used different aspiration techniques, and 47 did not have recorded aspiration tissue lengths, ultimately leaving 63 patients for analysis of aspiration technique. The process is depicted in Supplementary Figure S1. Ultimately, 90 out of 117 (52.6%) patients were categorized in the 19G group and 81 out of 171 (47.4%) in the 22G group; 34 out of 63 (54.0%) patients were in the SS group and 29 out of 63 (46.0%) in the HWS group. There were no significant differences in age, gender, lesion location, lesion size, platelet count, or prothrombin index between the needle puncture or aspiration technique groups (Tables 1, 2). Based on the final diagnosis, a total of 171 lesions were finally diagnosed based on pathologic evaluation and follow-up data, and only 36/171 (21.1%) patients underwent surgery. The final diagnosis showed that pancreatic carcinoma (86/171, 50.3%) was dominant in all cases, followed by chronic pancreatitis (22/171, 12.9%), metastatic carcinoma (22/171, 12.9%), neuroendocrine tumor (10/171, 5.8%), IgG4-related pancreatitis (6/171, 3.5%), mucinous cystadenoma (5/171, 2.9%), adenosquamous carcinoma (4/171, 2.3%), solid pseudopapillary neoplasm (4/171, 2.3%), serous cystadenoma (4/171, 2.3%), lymphoma (3/171, 1.8%), pancreatic tuberculosis (3/171, 1.8%), and mesenchymal tumor (2/171, 1.2%) (Table 3).

**Table 1:** Baseline characteristics and final diagnosis for puncture needle type.

	Procedure		*p*-value
	19G FNA	22G FNB	
Age, mean ± SD, y/o	60.88 ± 9.47	58.17 ± 11.23	0.090
Sex, *n* (%)			0.212
Male	50 (55.6)	53 (65.4)	
Female	40 (44.4)	28 (34.6)	
Location			0.240
Head and neck, *n* (%)	40 (44.4)	46 (56.8)	
Body and tail, *n* (%)	45 (50.0)	30 (37.0)	
Diffuse involvement, *n* (%)	5 (5.6)	5 (6.2)	
Size of the lesion, mm			0.339
<20 mm	27 (30.3)	17 (21.0)	
20–50 mm	42 (47.2)	41 (50.6)	
>50 mm	20 (22.5)	23 (28.4)	
Platelet count (/mm^3^)	301603.844 ± 93305.389	305385.444 ± 82181.061	0.791
Prothrombin index	1.004 ± 0.115	0.992 ± 0.112	0.480
Diagnosis following EUS-TA, *n* (%)			0.437
Pancreatic carcinoma	44 (48.9)	33 (40.7)	
Metastatic carcinoma	9 (10.0)	8 (9.9)	
Chronic pancreatitis	6 (6.7)	9 (11.1)	
Neuroendocrine tumor	5 (5.6)	5 (6.2)	
IgG4-related pancreatitis	3 (3.3)	2 (2.5)	
Mucinous cystadenoma	2 (2.2)	3 (3.7)	
Adenosquamous carcinoma	3 (3.3)	1 (1.2)	
Solid pseudopapillary neoplasm	3 (3.3)	1 (1.2)	
Serous cystadenoma	4 (4.4)	0 (0.0)	
Lymphoma	2 (2.2)	1 (1.2)	
Pancreatic tuberculosis	1 (1.1)	2 (2.5)	
Mesenchymal tumor	0 (0.0)	2 (2.5)	
Non-diagnostic	8 (8.9)	14 (17.3)	

Final diagnosis	19G FNA	22G FNB
Pancreatic carcinoma	47 (52.2)	39 (48.1)
Pancreatitis	12 (13.3)	16 (19.8)
Metastatic carcinoma	11 (12.2)	11 (13.6)
Neuroendocrine tumor	5 (5.6)	5 (6.2)
Mucinous cystadenoma	2 (2.2)	3 (3.7)
Adenosquamous carcinoma	3 (3.3)	1 (1.2)
Solid pseudopapillary neoplasm	3 (3.3)	1 (1.2)
Lymphoma	2 (2.2)	1 (1.2)
Serous cystadenoma	4 (4.4)	0 (0.0)
Pancreatic tuberculosis	1 (1.1)	2 (2.5)
Mesenchymal tumor	0 (0.0)	2 (2.5)

**Table 2:** Baseline characteristics and final diagnosis for suction technology.

	Procedure		*p*-value
	SS group	HWS group	
Age, mean ± SD, y/o	59.29 ± 11.71	56.52 ± 10.16	0.323
Sex, *n* (%)			0.199
Male	19 (55.9)	21 (72.4)	
Female	15 (44.1)	8 (27.6)	
Location			0.282
Head and neck, *n* (%)	14 (41.2)	15 (51.7)	
Body and tail, *n* (%)	19 (55.9)	11 (37.9)	
Diffuse involvement, *n* (%)	1 (2.9)	3 (10.3)	
Size of the lesion, mm			0.948
<20 mm	12 (35.3)	9 (31.0)	
20–50 mm	15 (44.1)	13 (44.8)	
>50 mm	7 (20.6)	7 (24.1)	
Puncture needle			1.000
19G FNA	18 (52.9)	16 (55.2)	
22G FNB	16 (47.1)	13 (44.8)	
Platelet count (/mm^3^)	292298.441 ± 88443.911	276241.517 ± 83619.566	0.464
Prothrombin index	1.030 ± 0.108	0.977 ± 0.101	0.051
Diagnosis following EUS-TA, *n* (%)			0.208
Pancreatic carcinoma	8 (23.5)	9 (31.0)	
Chronic pancreatitis	3 (8.8)	4 (13.8)	
Metastatic carcinoma	3 (8.8)	7 (24.1)	
Neuroendocrine tumor	4 (11.8)	3 (10.3)	
Mucinous cystadenoma	3 (8.8)	0 (0.0)	
Adenosquamous carcinoma	3 (8.8)	0 (0.0)	
Solid pseudopapillary neoplasm	2 (5.9)	1 (3.4)	
Lymphoma	1 (2.9)	1 (3.4)	
Serous cystadenoma	3 (8.8)	0 (0.0)	
IgG4-related pancreatitis	0 (0.0)	2 (6.9)	
Non-diagnostic	4 (11.8)	2 (6.9)	

**Table 3:** Final diagnosis for needle type.

Final diagnosis	19G FNA (*n* = 90)	22G FNB (*n* = 81)
Pancreatic carcinoma	47 (52.2)	39 (48.1)
Chronic pancreatitis	9 (10.0)	13 (16.0)
Metastatic carcinoma	11 (12.2)	11 (13.6)
Neuroendocrine tumor	5 (5.6)	5 (6.2)
IgG4-related pancreatitis	3 (3.3)	3 (3.7)
Mucinous cystadenoma	2 (2.2)	3 (3.7)
Adenosquamous carcinoma	3 (3.3)	1 (1.2)
Solid pseudopapillary neoplasm	3 (3.3)	1 (1.2)
Serous cystadenoma	4 (4.4)	0 (0.0)
Lymphoma	2 (2.2)	1 (1.2)
Pancreatic tuberculosis	1 (1.1)	2 (2.5)
Mesenchymal tumor	0 (0.0)	2 (2.5)

### 3.2 Comparison of puncture results between 19G FNA and 22G FNB needles

#### 3.2.1 Cytological diagnosis outcomes

In terms of cytological diagnosis, there was no statistically significant difference in the diagnostic rates between the 19G group and the 22G group (72.2% vs. 65.4%, *p* = 0.338). This suggests that the type of needle does not impact the cytological diagnosis outcomes (Table 4).

**Table 4:** Comparison of puncture result between 19G FNA and 22G FNB groups.

Item	19G FNA (*n* = 90)	22G FNB (*n* = 81)	Total (*n* = 171)	*p*-value
Accuracy of cytology, *n* (%)	65 (72.2)	53 (65.4)	118 (69.0)	0.338
Accuracy of histology, *n* (%)				
First pass	79 (87.8)	57 (70.4)	136 (79.5)	0.005
Second pass	74 (82.2)	53 (65.4)	127 (74.3)	0.012
First and second pass	81 (90.0)	59 (72.8)	140 (81.9)	0.004
Third pass	45 (50.0)	47 (58.0)	92 (53.8)	0.291
Final histology diagnosis	82 (91.1)	64 (79.0)	146 (85.4)	0.025
Accuracy of cytology and histology, *n* (%)	82 (91.1)	67 (82.7)	149 (87.1)	0.102
Third pass performed, *n* (%)	61 (67.8)	72 (88.9)	133 (77.8)	0.002
Procedure time, (minutes)	31.86 ± 4.13	34.46 ± 5.07	33.09 ± 4.77	<0.001
Adverse reaction, *n* (%)	7 (7.8)	5 (6.2)	12 (7.0)	0.770

#### 3.2.2 Histological diagnosis outcomes

Regarding histological diagnosis, the 19G group showed a higher definitive diagnosis rate on the first pass (87.8% vs. 70.4%, *p* = 0.005), the second pass (82.2% vs. 65.4%, *p* = 0.012), and the first two passes combined (88.9% vs. 72.8%, *p* = 0.004) compared to the 22G group. Through MOSE, a greater number of patients in the 22G group required a third pass compared to the 19G group (67.8% vs. 88.9%, *p* = 0.002), with both groups showing lower definitive diagnosis rates when only the third pass was considered. After the third needle pass, the definitive diagnosis rate in the 19G group remained higher than that in the 22G group (91.1% vs. 79.0%, *p* = 0.025) (Table 4). The total procedure time was shorter in the 19G group than in the 22G group (31.86 ± 4.13 min vs. 34.46 ± 5.07 min, *p* < 0.001). EUS-TA-related factors for the histological diagnostic accuracy were assessed using univariate and multivariate logistic regression (Table 5). In both analyses, needle sizes of EUS-TA (19G vs. 22G: OR 2.723 (1.105, 6.708), *p* = 0.029; OR 2.528 (1.015, 6.299), *p* = 0.046, respectively) were related to EUS-TA diagnosis accuracy. The lesion location (Head and neck as the control group: OR 2.407 (1.046, 5.537), *p* = 0.039) was only significant in the univariate analysis (Table 5).

**Table 5:** Uni- and multi-variable logistic analyses of factors associated with histological diagnosis accuracy of EUS-TA.

	Univariate analysis		Multivariate analysis	
	OR (95% CI)	*p*	OR (95% CI)	*p*
Age	0.960 (0.919, 1.004)	0.072	—	—
Male sex	0.815 (0.346, 1.920)	0.64	—	—
Location^a^	2.407 (1.046, 5.537)	0.039	2.229 (0.968, 5.132)	0.060
Size^b^	0.950 (0.721, 1.250)	0.712	—	—
Needle passes	0.627 (0.201, 1.954)	0.421	—	—
Needle size (19G vs. 22G)	2.723 (1.105, 6.708)	0.029	2.528 (1.015, 6.299)	0.046

a Head and neck as the control group.

b Size <2 cm as the control group.

#### 3.2.3 Cytological and histological combined diagnosis outcomes

**Figure 4:**
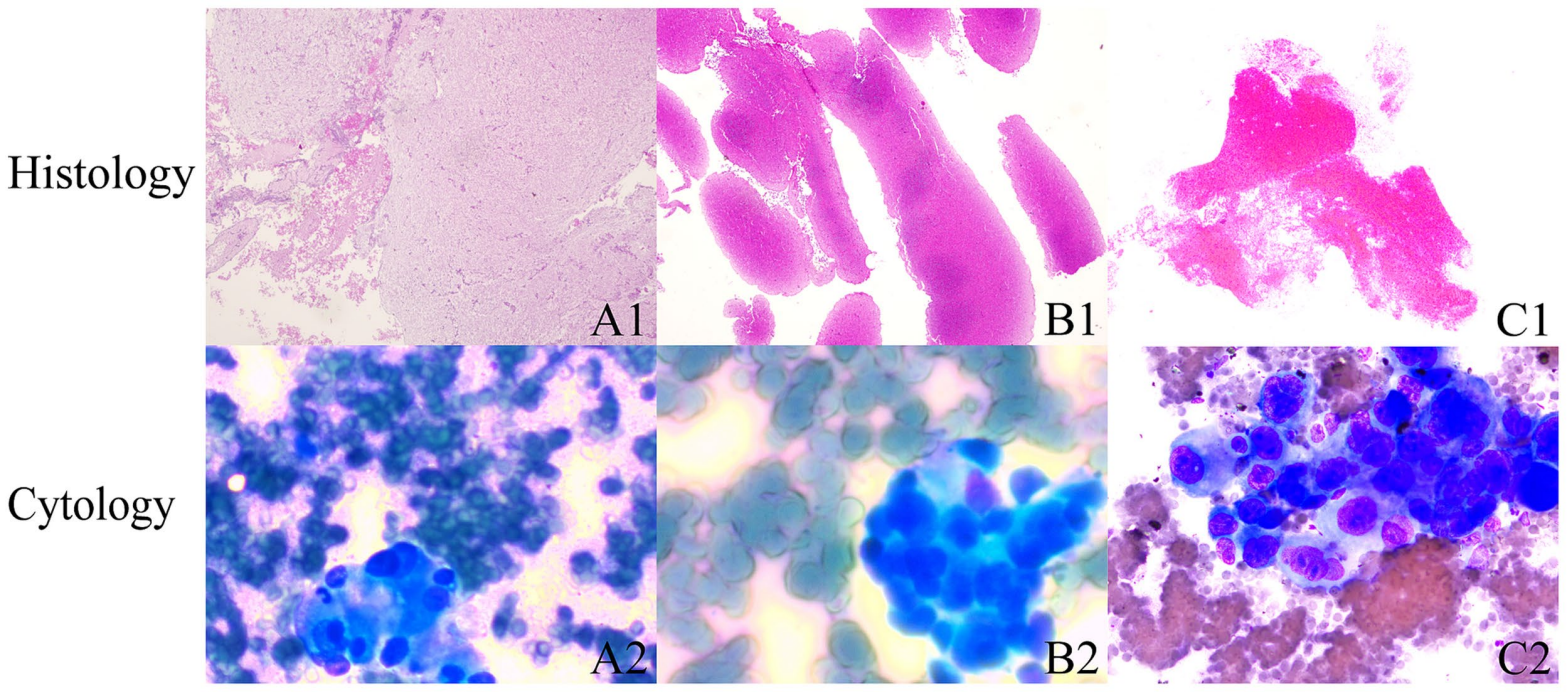
Cytology and histology diagnoses are inconsistent. **(A1–C1)** Shows that microscopic examination primarily reveals hemorrhage and fibrinous material, with scattered lymphocytes intermixed. **(B1,B2)** Indicate that microscopic examination primarily shows hemorrhage, with scattered lymphocytes intermixed. (HE stain; magnification, ×25). **(A2–C2)** Cytology: tumor cell clusters were observed within the hemorrhagic tissue. The tumor cells are round to oval, exhibiting an epithelial-like appearance, larger than five lymphocytes in size, with prominent nuclei and multiple nucleoli, and demonstrate strong adhesion.

In terms of the combined cytological and histological diagnosis, there was no statistically significant difference between the 19G group and the 22G group (91.1% vs. 82.7%, *p* = 0.102) (Table 4). This indicates that the type of needle does not affect the rate of combined cytological and histological diagnosis. However, the rate of definitive diagnosis using both cytological and histological analysis (87.1%) was higher than that of cytology alone (69.0%) or histology alone (85.4%) (Table 4). Notably, 3 patients (all from the 22G group) had a definitive cytological diagnosis, while their histopathological findings were defined as “undiagnosed” (Figure 4).

### 3.3 Comparison between SS and HWS techniques

#### 3.3.1 Length of tissue strips from punctures

Compared to the SS technique, the HWS group obtained longer total lengths of tissue strips from three passes (*p* < 0.001). The length of tissue strips from the first pass in the HWS group was longer than that in the SS group (*p* < 0.001) (Table 6).

**Table 6:** Comparison of puncture result between SS and HWS groups.

	SS group	HWS group	*p*-value
The length of tissue strips			
Total length of tissue strips (mm) (mean ± SD)	406.65 ± 45.28	464.79 ± 52.92	<0.001
The length of tissue at the first puncture (mm) (M, P25, P75)	136.00 (125.00, 156.25)	181.00 (168.50, 197.00)	<0.001
The length of tissue at the second puncture (mm) (mean ± SD)	140.38 ± 19.30	151.10 ± 24.78	0.098
The length of tissue at the third puncture (mm) (mean ± SD)	125.26 ± 16.61	131.97 ± 17.86	0.128
The length of white tissue core			
Total length of white tissue core (mm) (mean ± SD)	53.05 ± 15.41	62.83 ± 10.48	0.005
The length of white tissue core at the first puncture (mm) (M, P25, P75)	26.50 (19.00, 35.25)	34.00 (25.50, 39.50)	0.009
The length of white tissue core at the second puncture (mm) (M, P25, P75)	14.50 (11.00, 20.50)	17.00 (15.00, 21.00)	0.084
The length of white tissue core at the third puncture (mm) (M, P25, P75)	9.00 (8.00, 10.25)	10.00 (8.00, 14.50)	0.079
Adverse reaction (*n*, %)	2 (5.9)	3 (10.3)	0.654

#### 3.3.2 Length of white core tissue strips

In the HWS group, the length of the white core tissue obtained from the first pass was longer than that from the second or third pass. Compared to the SS group, the total length of white core tissue obtained from three passes was longer in the HWS group (*p* = 0.005), and the length of the white core tissue from the first pass in the HWS group was longer than that in the SS group (*p* = 0.009) (Table 6).

#### 3.3.3 Histological integrity

**Figure 5:**
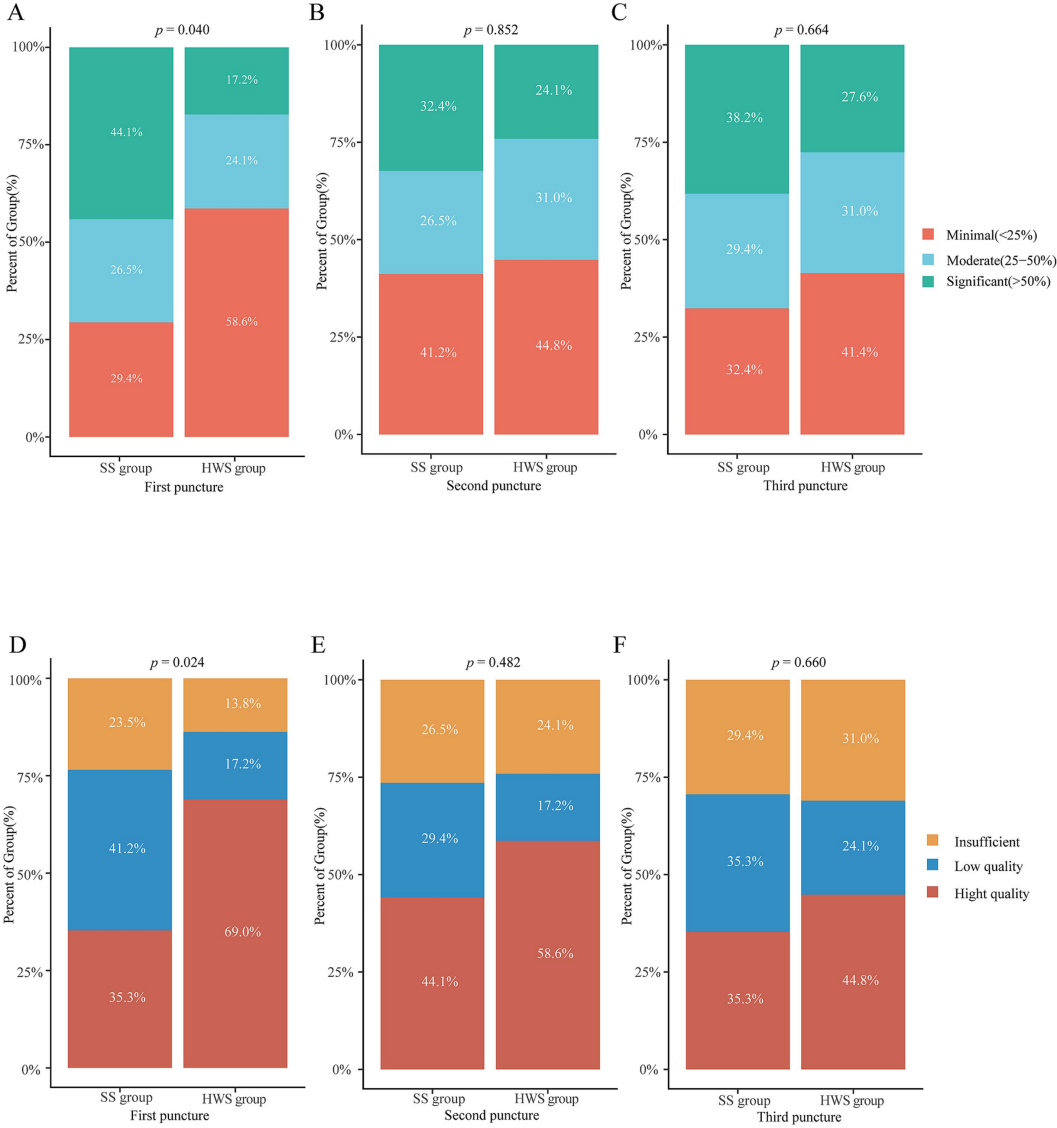
Comparison of the grading of blood cell contamination and histological integrity between the SS and HWS groups. **(A–C)** Blood cell contamination. **(D–F)** Histological integrity.

The histological integrity of the first needle pass in the HWS group was superior to that of the SS group (*p* = 0.024). There were no statistically significant differences in the integrity of the samples from the second (*p* = 0.482) and third (*p* = 0.660) passes between the two groups (Figure 5).

#### 3.3.4 Blood cell contamination grading

The grading of blood cell contamination in the first needle pass was better in the HWS group compared to the SS group (*p* = 0.040). There were no statistically significant differences in blood cell contamination grading for the second (*p* = 0.852) and third (*p* = 0.664) passes between the two groups (Figure 5).

#### 3.3.5 Diagnosis yield

The final diagnosis for suction method was displayed in Supplementary Table S2. In cytological diagnosis, there was no statistically significant difference in diagnostic rates between the SS and HWS groups (*p* = 0.231). In terms of histological diagnosis, there were no differences in the diagnostic rates for the first pass (*p* = 0.215), second pass (*p* = 0.832), third pass (*p* = 0.889), the first and second passes combined (*p* = 0.642), and the first three passes combined (*p* = 0.561) between the SS and HWS groups. In terms of the combined cytological and histological diagnosis, the diagnostic rates of the SS group and the HWS group were comparable (*p* = 0.308) (Supplementary Table S3).

### 3.4 Complications and follow-up

During the short-term follow-up period, intermittent mild upper abdominal pain was observed in 12 patients, with 7 cases originating from the 19G group and 5 from the 22G group, showing no statistically significant difference (*p* = 0.770). Additionally, 3 cases were from the HWS group and 2 from the SS group, also with no statistically significant difference (*p* = 0.654) (Tables 4, 6). Additionally, we conducted clinical follow-ups at 6 months after patient discharge, and no long-term complications were observed in any of the patients.

## 4 Discussion

With the rise of personalized medicine, it is now necessary to obtain more tissue for next-generation sequencing, molecular analysis, and organoid generation (1, 19). Since ROSE is costly, time-consuming, and often unavailable in many centers (22, 25), the selection of puncture needles and aspiration technique has become a primary factor in obtaining sufficient specimens in EUS-TA (4, 24, 26). Our study demonstrates that, in terms of histological diagnosis rates for SPLs, the 19G FNA needle outperforms the 22G ProCore FNB needle. With MOSE, the 19G group required fewer needle passes to complete EUS-TA, resulting in a shorter total procedure time compared to the 22G group. The combined cytological and histological evaluation was superior to either cytology or histology alone. The HWS method, compared to the SS method, can enhance the volume and integrity of tissue samples, reduce blood cell contamination, and demonstrates a high level of safety.

### 4.1 19G FNA needle superior to 22G FNB needle

EUS-TA can provide both cytological and histological results, with the latter being more valuable. Lee and Kim (1) had indicated that if core tissue is needed, FNB needles or 19G FNA needles should be considered. ESGE recommends using 19G FNA needles, 19G FNB needles, or 22G FNB needles for tissue specimen acquisition (17). A meta-analysis revealed no significant differences in diagnostic adequacy (75.2% vs. 89.0%; *p* = 0.23), diagnostic accuracy (85.8% vs. 86.2%; *p* = 0.53), or histological core specimen acquisition (77.7% vs. 76.5%; *p* = 0.85) between the 19G ProCore needles and EUS-FNA needles (15). Additionally, the 19G FNB needles were associated with a higher incidence of adverse events (10). Therefore, our study compared 19G FNA needles with 22G FNB needles in SPLs.

Our study indicates that the type of needle tip does not affect the cytological diagnosis outcomes for SPLs. Regarding histological diagnosis, the 19G group showed significantly higher definitive diagnosis rates compared to the 22G group. Through MOSE, most patients in the 19G group only require 1–2 passes to achieve a high diagnostic rate, whereas the 22G group might need a third pass. This could suggest that the 19G FNA needle obtains more core tissue and that operators can easily detect this core tissue when using the 19G FNA needle. Furthermore, comprehensive genomic profiling (CGP) demands a substantial quantity of specimens. The larger the diameter of the needle, the higher the success rate of the analysis (8). Therefore, endoscopists should consider using the 19G FNA needle judiciously, as the risk of complications may increase with the number of punctures (8, 27). Moreover, needle tip bending and displacement of the target tissue due to repeated punctures could limit the accuracy gains obtained by increasing the number of punctures (28). Additionally, the 19G FNA needle demonstrated superior performance compared to the 22G FNB needle by shortening the overall procedure time. Finally, FNB needles are typically more expensive than FNA needles. In practical settings, these benefits significantly influence the operational efficiency of an endoscopy center and the cost associated with each procedure.

Regarding the combined cytological and histological evaluation, there was no statistically significant difference in the diagnostic rates between the 19G group and the 22G group. Compared to cytology alone or histology alone, the combination of both methods increased the definitive diagnosis rate for SPLs, which is consistent with findings from previous studies (9). It is noteworthy that all three patients diagnosed through cytology came from the 22G group. The main reason for this is that lesions interpreted as “inconclusive” by histopathology have a higher degree of malignancy, with necrosis in the central part of the tumor and severe interstitial fibrous encapsulation. This leads to a lack of effective puncture tissue or insufficient tissue quantity, yet cytology has detected cancer cells in all cases (29). This phenomenon further suggests that the 19G group may more easily obtain effective lesion tissue; the examination methods of cytology combined with histopathology can complement each other, thereby better improving the diagnostic efficacy of SPLs. For cases where the histopathology result is “inconclusive” but clinically suspected to be malignant, it is recommended to perform a repeat biopsy to reduce misdiagnosis and missed diagnosis caused by improper sampling location.

### 4.2 HWS technique superior to SS technique

A previous study (21) demonstrated that the HWS technique yielded longer white core tissue in the first pass and had a lower overall rate of blood contamination compared to the SS technique. However, that study included only 50 cases and did not compare the integrity of the specimens between the two groups. Complete tissue samples are particularly important for lesions requiring histomorphological identification (24). Our study shows that the tissue integrity from the first pass in the HWS group was superior to that in the SS group. In terms of tissue sample length, the HWS group also outperformed the SS group. The possible reasons for these results include heparin preventing tissue strips from sticking to the needle wall due to blood coagulation, thereby protecting sample integrity while increasing the sample volume. Furthermore, the HWS group pre-fills the needles with heparinized saline, replacing air with liquid, which allows the negative pressure applied by the syringe to be more effectively transmitted to the needle tip (30). Finally, due to fluid dynamics, the suction generated by HWS technology exceeds that of standard technology (31). Better suction leads to a larger volume of sample being aspirated; consequently, the longer the tissue strip is, the longer the segment of white core tissue becomes. There were no statistically significant differences in histological integrity between the two groups for the second and third punctures. We speculate this may be because the SS group used a dry needle for the first puncture. During the second and third punctures, rinsing the needle lumen with saline wet the needle lumen (similar to the WS technique), and the saline film on the wet needle wall could reduce the friction between the attractants and the needle wall, allowing the sample inside the needle to move more smoothly, thereby improving sample integrity. We found that the lengths of tissue strips and white core tissue strips from the latter two punctures did not significantly differ between the two groups, confirming our speculation.

Regarding the grading of smear blood cell contamination, our study found that the first pass in the HWS group had a better blood cell contamination grade than the SS group. Considering heparin’s anticoagulant properties, an increase in blood cell contamination rate was anticipated. In reality, due to capillary action, heparin remained within the needle lumen and did not permeate into the target tissue (21). Additionally, the wet suction technique facilitated faster aspiration of lesion tissue into the needle’s tip, while the presence of heparin saline eliminated the “gaps” in the needle lumen through which red blood cells could flow, thereby reducing the sample’s blood contamination rate (32). It is generally believed that larger and more intact samples not only meet the standards for pathological diagnosis but can also be used for auxiliary examinations such as IHC staining (33, 34), leading to better diagnostic outcomes. With the advancement of precision medicine, a larger sample volume is advantageous for next-generation sequencing (35). Therefore, we recommend using the HWS technique for the first puncture to improve specimen quality.

### 4.3 Adverse events

It is commonly believed that thicker needles may not perform satisfactorily when puncturing certain specific locations, such as the duodenum (8). However, studies have confirmed the feasibility and accuracy of using 19G needles to obtain samples from the duodenum (36). Regarding the anticoagulant action of heparin, adverse events were anticipated to increase but have been proven safe (20, 37). During a short-term follow-up period, intermittent mild upper abdominal pain was observed in 12 patients, there was no significant difference in complication rates between the needle puncture groups or the aspiration technique groups. All 12 patients were successfully discharged after a 3-day infusion of proton pump inhibitors. No further adverse events were reported.

## 5 Limitations

Our study has several limitations. First, we were unable to access newer needle types such as SharkCore or Franseen in recent years, hence this study did not compare the 19G FNA needle with various types of needles, which necessitates further research. Second, we analyzed needle type and suction method separately due to the lack of recorded aspiration tissue lengths in earlier puncture samples. The limited sample size from the extraction method restricted our ability to divide samples into “four-branch” groups (19G-SS, 19G-HWS, 22G-SS, 22G-HWS) for a more robust study design. Third, our center lacks ROSE; however, studies indicate that MOSE seems to be a viable alternative to ROSE (5, 38). In this study, the endoscopist was trained prior to the trial in MOSE and assessment of sample sufficiency.

## 6 Conclusion

In summary, EUS-FNA with a 19G needle enables the collection of adequate tissue specimens, resulting in higher histological diagnostic accuracy with fewer needle passes and a shorter total procedure time. In addition, compared to the SS technique, the HWS technique for the first pass is recommended to improve specimen quality. Future studies should involve larger samples and assess whether tissues obtained using the 19G FNA needle and HWS technique are adequate for molecular analysis and precision treatment of cancer.

## Data Availability

The raw data supporting the conclusions of this article will be made available by the authors, without undue reservation.
