# Supplementary material for: Comparing needle types and aspiration techniques in EUS-TA to optimize diagnostic efficacy and specimen quality in patients with pancreatic lesions
Source: Front Med (Lausanne). 2024 Dec 6;11:1422600. doi: 10.3389/fmed.2024.1422600 (PMC11658985; doi:10.3389/fmed.2024.1422600)
Supplement: Supplementary file 2 [file Table_2.docx]

**Table. S2.** Final Diagnosis for sunction technology.

| **Final diagnosis** | SS (n= 34) | HWS (n=29) |
| --- | --- | --- |
| Pancreatic carcinoma | 11 (32.4) | 9（31.0） |
| Metastatic carcinoma | 4 (11.8) | 8 (27.6) |
| Chronic pancreatitis | 3 (8.8) | 5 (17.2) |
| Neuroendocrine tumor | 4(11.8) | 3 (10.3) |
| Mucinous cystadenoma | 3 (8.8) | 0 (0.0) |
| Adenosquamous carcinoma | 3 (8.8) | 0 (0.0) |
| Solid pseudopapillary neoplasm | 2 (5.9) | 1 (3.4) |
| Serous cystadenoma | 3 (8.8) | 0 (0.0) |
| IgG4-related pancreatitis | 0 (0) | 2 (6.9) |
| Lymphoma | 1 (2.9) | 1 (3.4) |
