# Supplementary material for: Comparing needle types and aspiration techniques in EUS-TA to optimize diagnostic efficacy and specimen quality in patients with pancreatic lesions
Source: Front Med (Lausanne). 2024 Dec 6;11:1422600. doi: 10.3389/fmed.2024.1422600 (PMC11658985; doi:10.3389/fmed.2024.1422600)
Supplement: Supplementary file 3 [file Table_3.docx]

### Table. S3. Comparison of diagnostic performance between SS and HWS groups.

| **Item** | **SS group**  **(n= 34)** | **HWS group (n= 29)** | **Total**  **(n= 63)** | ***P* value** |  |
| --- | --- | --- | --- | --- | --- |
| **Accuracy of cytology, n (%)** | 21 (61.8) | 22 (75.9) | 43 (68.3) | 0.231 | |
| **Accuracy of histology, n (%)** |  |  |  |  | |
| First pass | 25 (73.5) | 25 (86.2) | 50 (79.4) | 0.215 | |
| Second pass | 25 (73.5) | 22 (75.9) | 47 (74.6) | 0.832 | |
| First and second passes | 28 (82.4) | 26 (89.7) | 54 (85.7) | 0.642 | |
| Third pass | 24 (70.6) | 20 (69.0) | 44 (25.7) | 0.889 | |
| Final three passes | 29 (85.3) | 27 (93.1) | 56 (88.9) | 0.561 | |
| **Accuracy of cytology and histology, n (%)** | 30 (88.2) | 27 (93.1) | 57 (90.5) | 0.822 | |
