# Supplementary figures and images for: Comparing needle types and aspiration techniques in EUS-TA to optimize diagnostic efficacy and specimen quality in patients with pancreatic lesions
Source: Front Med (Lausanne). 2024 Dec 6;11:1422600. doi: 10.3389/fmed.2024.1422600 (PMC11658985; doi:10.3389/fmed.2024.1422600)

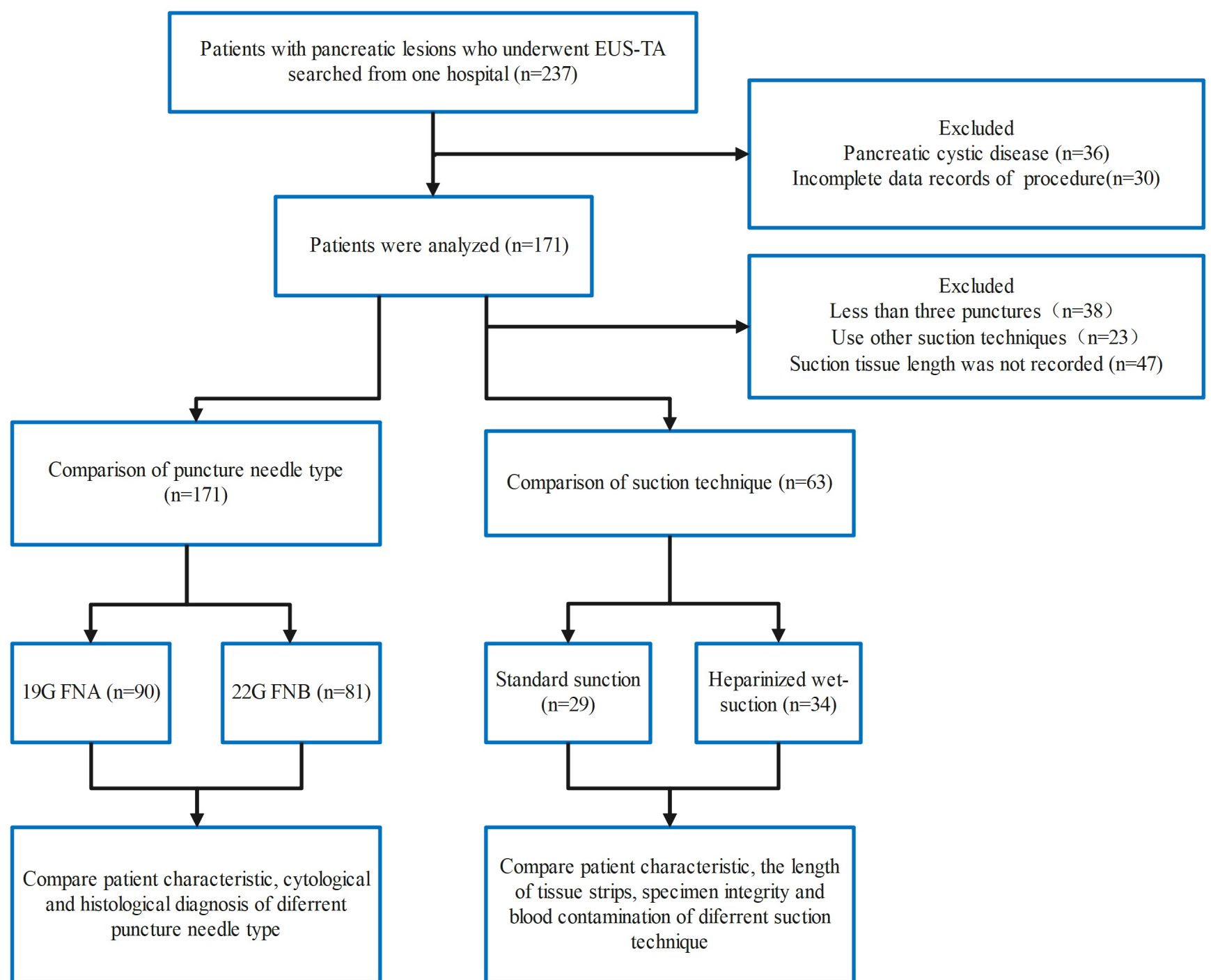

FigureS1 The flow chart of this study

Supplement: Supplementary file 4 [file Data_Sheet_1.PDF]
